# COVID-19 mRNA vaccination status and concerns among pregnant women in Japan: a multicenter questionnaire survey

**DOI:** 10.1186/s12884-023-05669-4

**Published:** 2023-05-09

**Authors:** Ken Takahashi, Osamu Samura, Akihiro Hasegawa, Haruna Okubo, Keiji Morimoto, Madoka Horiya, Aikou Okamoto, Daigo Ochiai, Mamoru Tanaka, Masaki Sekiguchi, Naoyuki Miyasaka, Yuto Suzuki, Tsutomu Tabata, Eijiro Hayata, Masahiko Nakata, Tomoo Suzuki, Hirotaka Nishi, Yumi Toda, Shinji Tanigaki, Natsumi Furuya, Junichi Hasegawa, Shunsuke Tamaru, Yoshimasa Kamei, Seisuke Sayama, Takeshi Nagamatsu, Yuka Otera Takahashi, Michihiro Kitagawa, Tatsuya Arakaki, Akihiko Sekizawa

**Affiliations:** 1 Department of Obstetrics and Gynecology, The Jikei University Hospital, 3-19-18 Nishi-Shimbashi, Minato-Ku, Tokyo, 105-8471 Japan; 2 Department of Obstetrics and Gynecology, The Jikei University School of Medicine, 3-25-8 Nishi-Shimbashi, Minato-Ku, Tokyo, 105-8461 Japan; 3 Department of Obstetrics and Gynecology, The Jikei University Katsushika Medical Center, 6-41-2 Aoto, Katsushika-Ku, Tokyo, 125-8506 Japan; 4 Department of Obstetrics and Gynecology, The Jikei University Daisan Hospital, 4-11-1 Izumihonmachi, Komae, Tokyo, 201-8601 Japan; 5 Department of Obstetrics and Gynecology, The Jikei University Kashiwa Hospital, 163-1 Kashiwashita, Kashiwa City, Chiba 277-8567 Japan; 6 Department of Obstetrics and Gynecology, Keio University Hospital, 35 Shinanomachi, Shinjuku-Ku, Tokyo, 160-8582 Japan; 7 Department of Obstetrics and Gynecology, Tokyo Medical and Dental University, 1-5-45 Yushima, Bunkyo-Ku, Tokyo, 113-8519 Japan; 8 Department of Obstetrics and Gynecology, Tokyo Women’s Medical University, 8-1 Kawadacho, Shinjuku, Tokyo, 162-8666 Japan; 9 Department of Obstetrics and Gynecology, Toho University Omori Medical Center, 6-11-1 Omorinishi, Ota-Ku, Tokyo, 143-8541 Japan; 10 Department of Obstetrics and Gynecology, Tokyo Medical University, 6-7-1 Nishi-Shinjuku, Shinjuku-Ku, Tokyo, 160-0023 Japan; 11 Department of Obstetrics and Gynecology, Kyorin University Hospital, 6-20-2 Shinkawa, Mitaka City, Tokyo, 181-8611 Japan; 12 Department of Obstetrics and Gynecology, St. Marianna University School of Medicine Hospital, 2-16-1 Sugao, Miyamae-Ku, Kawasaki City, Kanagawa, 216-8511 Japan; 13 Department of Obstetrics and Gynecology, Saitama Medical University Hospital, 38 Morohongo, Moroyama-Machi, Iruma-Gun, Saitama, 350-0495 Japan; 14 Department of Obstetrics and Gynecology, The University of Tokyo Hospital, 7-3-1 Hongo, Bunkyo-Ku, Tokyo, 113-8655 Japan; 15 Sanno Birth Center, 8-10-16 Akasaka, Minato-Ku, Tokyo, 107-0052 Japan; 16 Department of Obstetrics and Gynecology, Showa University School of Medicine, 1-5-8 Hatanodai, Shinagawa-Ku, Tokyo, 142-8666 Japan

**Keywords:** mRNA vaccine, SARS-CoV-2, Fetus, Safety, Perinatal outcomes

## Abstract

**Background:**

mRNA vaccination is an effective, safe, and widespread strategy for protecting pregnant women against infection with severe acute respiratory syndrome coronavirus 2 (SARS-CoV-2) infection. However, information on factors such as perinatal outcomes, safety, and coverage of mRNA vaccinations among pregnant women is limited in Japan. Therefore, this study aimed to investigate the perinatal outcomes, coverage, adverse effects, and short-term safety of mRNA vaccination as well as vaccine hesitancy among pregnant women.

**Methods:**

We conducted a multicenter online survey of postpartum women who delivered their offspring at 15 institutions around Tokyo from October 2021 to March 2022. Postpartum women were divided into vaccinated and unvaccinated groups. Perinatal outcomes, COVID-19 prevalence, and disease severity were compared between the two groups. Adverse reactions in the vaccinated group and the reasons for being unvaccinated were also investigated retrospectively.

**Results:**

A total of 1,051 eligible postpartum women were included. Of these, 834 (79.4%) had received an mRNA vaccine, while 217 (20.6%) had not, mainly due to concerns about the effect of vaccination on the fetus. Vaccination did not increase the incidence of adverse perinatal outcomes, including fetal morphological abnormalities. The vaccinated group demonstrated low COVID-19 morbidity and severity. In the vaccinated group, the preterm birth rate, cesarean section rate, and COVID-19 incidence were 7.2%, 33.2%, and 3.3%, respectively, compared with the 13.7%, 42.2%, and 7.8% in the unvaccinated group, respectively. Almost no serious adverse reactions were associated with vaccination.

**Conclusions:**

mRNA vaccines did not demonstrate any adverse effects pertaining to short-term perinatal outcomes and might have prevented SARS-CoV-2 infection or reduced COVID-19 severity. Concerns regarding the safety of the vaccine in relation to the fetus and the mother were the main reasons that prevented pregnant women from being vaccinated. To resolve concerns, it is necessary to conduct further research to confirm not only the short-term safety but also the long-term safety of mRNA vaccines.

**Supplementary Information:**

The online version contains supplementary material available at 10.1186/s12884-023-05669-4.

## Background

Coronavirus disease 2019 (COVID-19) during pregnancy is associated with severe maternal morbidity, mortality, and neonatal complications [1]. Vaccines against infectious diseases are crucial, given that mRNA vaccines such as BNT162b2 (Pfizer-BioNTech) and mRNA-1273 (Moderna) have reportedly reduced the risk of severe acute respiratory syndrome coronavirus 2 (SARS-CoV-2) infection and prevented the incidence of severe COVID-19 [2, 3]. Moreover, several studies and a recent meta-analysis have reported the safety and efficacy of mRNA vaccines in pregnant women [4–6].

However, given the differences in medical systems and national vaccination policies, vaccination coverage varies across different countries [7], especially in pregnant women [8]. In Japan, mRNA vaccination was initiated on February 17, 2021, with the Pfizer-BioNTech vaccine, and Moderna and AstraZeneca vaccines were introduced later. The Japan Society of Obstetrics and Gynecology recommends vaccination of all pregnant women [9]. However, mRNA vaccination of pregnant women is not compulsory in Japan and is based on individual preference. A survey of 202 pregnant women from a multi-ethnic population in north London reported that 56.9% of pregnant women refused to get the COVID-19 vaccine. Various factors, such as age and ethnicity, have been reported to influence the decision of getting vaccinated [10]. In Japan, more than 80% of the population has received at least one vaccination as of March 2022; however, information on the vaccination status, especially of pregnant women, is lacking [11]. Thus, the availability of information on vaccination and related issues in pregnant women in Japan is limited. The severity of COVID-19 varies according to the genetic factors of the human host and the SARS-CoV-2 variant [12]; therefore, it is necessary to collect data on the impact of COVID-19 on Japanese pregnant women.

In Japan, the trust of the public in the safety and effectiveness of vaccines is low because of the history of the onset of functional somatic syndromes in young Japanese women who had received the human papillomavirus vaccination [13, 14]. Hence, we hypothesized that mRNA vaccination during pregnancy may be less acceptable to pregnant women in Japan than in other countries, owing to concerns about the effect of vaccination on the fetus. Therefore, this study aimed to examine the perinatal outcomes, coverage, adverse effects, and short-term safety of mRNA vaccination among pregnant women in Japan as primary outcomes and vaccine hesitancy as a secondary outcome.

## Methods

### Study design and data source

We conducted a multicenter cross-sectional survey of Japanese women in their postpartum period within 1 month. The participants were enrolled from 15 institutions, which were mainly located at university hospitals around Tokyo, Japan (Additional file 1), and are experienced in managing high-risk pregnant women. The women were enrolled from October 2021 to March 2022 during the COVID-19 pandemic. The inclusion criteria for the study were as follows: pregnant women aged ≥ 20 years who had consented to participate in the study and who underwent delivery management, including stillbirth at a participating facility during the study period. Participants who did not meet the inclusion criteria, who did not respond to the vaccination questions, or who were vaccinated before the current pregnancy were excluded. A questionnaire containing simple terms that were easy for non-medical individuals to understand was designed using the Japanese version of Google Forms. A QR code was attached to a leaflet describing the outline of the study, and the participants scanned the QR code on their smartphones to access the survey and provided their responses. The questionnaire contained a section on informed consent for participating in the study; it included questions about the patient’s background information, SARS-CoV-2 infection history, the perinatal outcome of pregnancy and newborns, mRNA vaccination status during the current pregnancy, vaccine type, adverse reactions, number of vaccine doses received, and reasons for not being vaccinated. Receiving two doses of the mRNA vaccine was the norm in Japan during the study period, but pregnant women who received at least one dose of the vaccine during the current pregnancy were assigned to the vaccinated group. The severity of COVID-19 was assessed according to the requirement for supplemental oxygen. Based on the mRNA vaccination status, the participants were divided into the following two groups: postpartum women who received mRNA vaccination during pregnancy (vaccinated group), and postpartum women who did not receive mRNA vaccination during pregnancy (unvaccinated group). The outcomes were compared between the two groups.

### Ethics approval

Informed consent was obtained from all individual participants included in the study. The study was approved by the Ethics Committee of the Jikei University School of Medicine (approval number: 33–108). This study was conducted in accordance with the ethical standards of the Declaration of Helsinki and the Japanese national ethical guidelines.

### Statistical analyses

After confirming normal data distribution, student’s t-test was used to analyze the continuous variables (age, height, and body weight), which are expressed as mean and standard deviation or median (interquartile range) or percentage. The χ^2^ test or Fisher’s exact tests were used to compare the proportions of categorical variables, including preterm birth rates, cesarean section rates, and past medical histories, between the two groups. Statistical significance was set at *p* < 0.05. For each question, participants with missing data were excluded when calculating percentages. Statistical analyses were performed using Excel spreadsheets (Microsoft Corporation, Redmond, WA, USA) or JMP 9.0.2 (SAS Institute Japan).

## Results

### Study population and participant characteristics

**Fig. 1:**
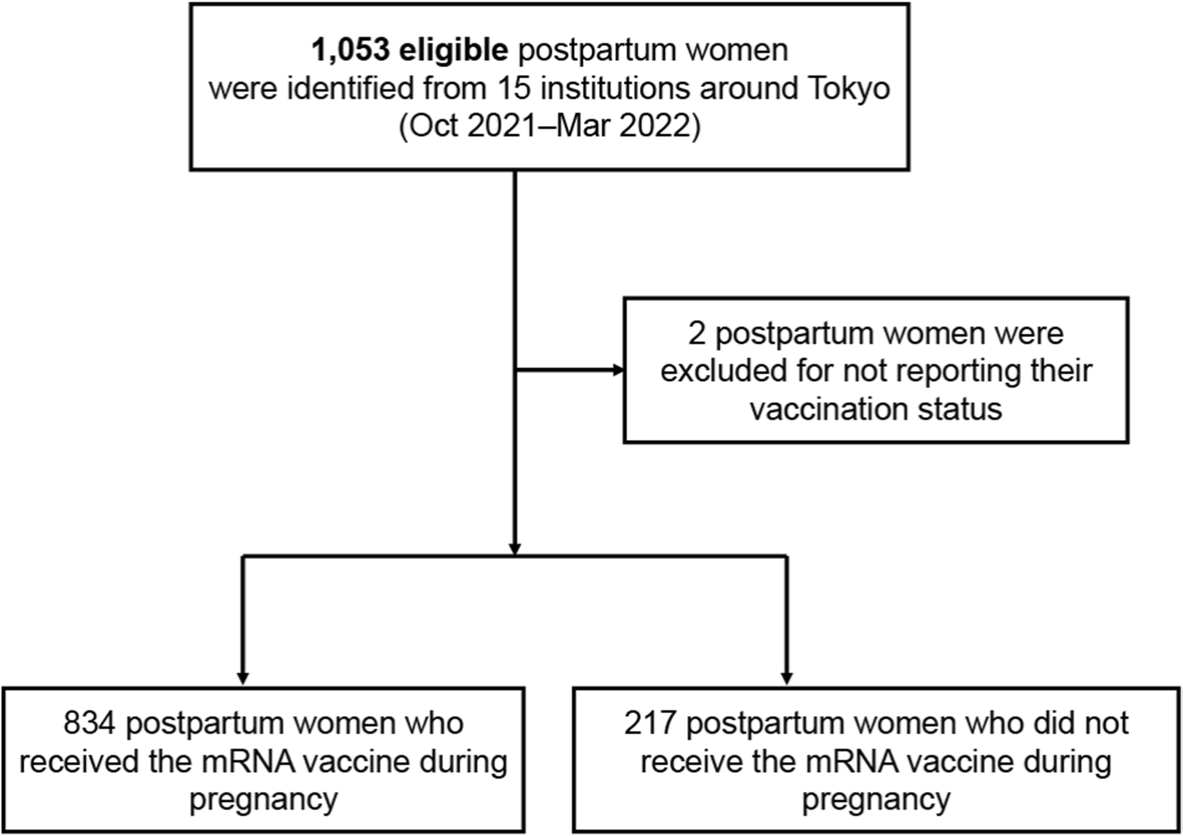
Study flow chart. Initially, 1,053 postpartum women were eligible. After exclusion of two women, a total of 1,051 women were finally examined in the study. Among these, 834 were vaccinated, and 217 were unvaccinated

A total of 1,053 eligible postpartum women who gave birth at the target institutions during the study period provided consent and were enrolled in the study. Two postpartum women were excluded from the analysis as they did not provide information on their vaccination status. Overall, 834 (79.4%) participants received an mRNA vaccine during pregnancy and 217 (20.6%) did not (Fig. 1). Of the 834 vaccinated participants, 830 (99.5%) received an mRNA vaccine [BNT162b2 (Pfizer-BioNTech) or mRNA-1273 (Moderna)], 3 (0.4%) did not know the type of vaccine that they had received, and only 1 (0.1%) received viral vector vaccine [ChAdOx1-S (AstraZeneca)].

Table 1 summarizes the participants’ characteristics. The only significant difference between the two groups was noted in the frequency of asthma, in that this frequency was significantly higher in the unvaccinated group than in the vaccinated group (14.8% vs. 10.1%, *p* = 0.049). The proportion of pregnant women with allergies was higher in the unvaccinated group than in the vaccinated group (14.8% vs. 10.7%, *p* = 0.098 for drug allergies; 16.6% vs. 14.2%, *p* = 0.369 for food allergies) but not at a statistically significant level.

**Table 1:** Participants background information

	Postpartum women vaccinated during pregnancy	Postpartum women not vaccinated during pregnancy	*p*-value*
Maternal age (years)	35 (31–38)	34 (31–37)	0.165
Gravidity (including the current pregnancy)	2 (1–2)	2 (1–2)	0.355
Parity (including the current pregnancy)	1 (1–2)	1 (1–2)	0.294
Natural pregnancy rate	68.6 (569/829)	70.5 (153/217)	0.596
Height (cm)	159.1 ± 5.5	158.7 ± 5.3	0.439
Pre pregnancy Weight (kg)	54.2 ± 9.9	53.8 ± 8.4	0.574
Smoking rate	3.0 (25/831)	4.1 (9/217)	0.399
Singleton pregnancy rate	97.1 (807/831)	95.7 (202/211)	0.308
**Past medical history**			
Asthma	10.1 (84/831)	14.8 (32/216)	0.049
Diabetes	9.9 (83/833)	13.0 (28/216)	0.202
Hypertension	8.5 (71/833)	7.4 (16/216)	0.596
Malignant disease	1.9 (16/833)	1.9 (4/216)	0.947
Autoimmune disease	2.9 (24/833)	4.17 (9/216)	0.335
**Allergy**			
Drug allergy	10.7 (89/831)	14.8 (32/217)	0.098
Food allergy	14.2 (118/833)	16.6 (36/217)	0.369

Data are shown as the mean ± standard deviation or median (interquartile range) or percentage (Number of applicable women/Number of women targeted for analysis). *Student's *t*-test, or χ2 test

The number of subjects is calculated by excluding the missing values from the total number of each group

### SARS-CoV-2 infection during pregnancy and timing of infection

Table 2 shows the occurrence of SARS-CoV-2 infections during pregnancy, maternal gestation at the time of infection, and vaccination status. The incidence of COVID-19 was significantly lower in the vaccinated group than in the unvaccinated group (3.3% vs. 7.8%, *p* = 0.003). The rate of oxygen administration for patients with COVID-19, which is an index of disease severity, was lower in the vaccinated group than in the unvaccinated group, although this difference was not statistically significant (3.7% vs. 11.7%, *p* = 0.550). The incidence of infection during the third trimester, a period when COVID-19-related pneumonia tends to be more severe [15], was similar between the two groups.

**Table 2:** Presence or absence of SARS-CoV2 infection and maternal infection time in gestation

	Postpartum women vaccinated during pregnancy	Postpartum women not vaccinated during pregnancy	*p-*value*
SARS-CoV-2 infection	3.3 (27/828)	7.8 (17/217)	0.003
Oxygen administration due to COVID-19	3.7 (1/27)	11.7 (2/17)	0.550
Percentage of maternal infections during pregnancy			
First trimester	11.1 (3/27)	11.8 (2/17)	
Second trimester	25.9 (7/27)	17.6 (3/17)	
Third trimester	62.9 (16/27)	58.8 (10/17)	
Unknown	0.0 (0/27)	11.8 (2/17)	

Data are shown as the percentage (number of applicable women/number of women targeted for analysis). *χ2 test or *Fisher's* exact test

The number of subjects is calculated by excluding the missing values from the total number of each group

### Perinatal outcome of pregnancy and newborns

Three miscarriages occurred in the vaccinated group, and six miscarriages occurred in the unvaccinated group. The participants who had miscarriages were excluded from the perinatal outcome analysis. The median gestational age at delivery was 38 weeks in both groups. Additionally, we analyzed the distribution of the percentage of deliveries in each gestational week for both the vaccinated and unvaccinated groups (Additional file 2); however, the preterm birth (7.2% vs. 13.7%, *p* = 0.002) and cesarean Sect. (33.2% vs. 42.2%, *p* = 0.015) rates were significantly lower in the vaccinated group than in the unvaccinated group (Table 3). When restricted to participants with COVID-19, the cesarean section rates for the vaccinated and unvaccinated groups were 50.0% (13/26) and 41.2% (7/17), respectively, and the preterm delivery rates were 0.0% (0/26) and 17.6% (3/17), respectively. No significant differences were noted in the singleton pregnancy rate, neonatal birth weight, or length at birth. The prevalence of neonatal congenital diseases, including malformations, in the vaccinated and unvaccinated groups, were 4.1% and 3.6%, respectively, with no significant difference between the two groups. No incidents of fetal death or neonatal death after 22 weeks in the vaccinated group.

**Table 3:** Perinatal outcomes in the mRNA-vaccinated and non-mRNA-vaccinated groups

	Postpartum women vaccinated during pregnancy	Postpartum women not vaccinated during pregnancy	*p* value*
Gestational weeks at delivery (Median)	38 (38–39)	38 (37–39)	
Preterm birth	7.2 (60/831)	13.7 (29/211)	0.002
Caesarean section	33.2 (276/831)	42.2 (89/211)	0.015
Neonatal Birth weight(g)	2904 ± 484	2845 ± 602	0.177
Neonatal Birth height (cm)	48.6 ± 2.7	48.2 ± 3.3	0.103
Congenital disease among all neonates	4.1 (35/855)	3.6 (8/220)	0.758

Data are shown as the number or mean ± standard deviation or median (interquartile range) or percentage (number of applicable women/number of women targeted for analysis). *Student's *t*-test, or χ2 test

The number of subjects is calculated by excluding the missing values from the total number of each group

### Timing and adverse effects of mRNA vaccination

The timing of vaccination was distributed throughout the gestational period among the 834 postpartum women in the vaccination group (Table 4). During the study period, 1.1% of the postpartum women received only one dose of vaccine during pregnancy. The incidence of adverse reactions was significantly higher for the second dose of the mRNA vaccine. However, no significant difference was noted in the serious adverse effects such as thrombosis and anaphylaxis between the first and second doses of vaccine, although the rate of requiring treatment tended to be higher with the second dose of vaccine than the first dose (0.37% vs. 0.12%, *p* = 0.311).

**Table 4:** Timing and adverse effects of mRNA vaccination

	First dose of vaccination %	Second dose of vaccination %	*p-*value*
**Inoculation timing**			
First trimester	18.7 (156/834)	8.2 (68/834)	
Second trimester	61.8 (515/834)	58.2 (485/834)	
Third trimester	18.1 (151/834)	31.4 (262/834)	
Unvaccinated	0.0 (0/834)	1.1 (9/834)	
Unknown	1.4 (12/834)	1.2 (10/834)	
**Adverse reactions (multiple answers)**			
Headache	18.5 (153/828)	33.4 (274/820)	< 0.001
Malaise	46.3 (383/828)	66.1 (543/822)	< 0.001
Dizziness	2.8 (23/828)	6.2 (51/819)	< 0.001
Fever (> 37.5 ℃)	12.7 (105/827)	33.0 (271/820)	< 0.001
Cold	8.2 (68/828)	23.9 (196/821)	< 0.001
Nausea	3.4 (28/828)	6.6 (54/820)	0.003
Pain at injection site	90.0 (745/828)	81.0 (665/821)	< 0.001
Breathing difficulty	0.61 (5/825)	17.1 (14/821)	0.037
Thrombosis	0.12 (1/825)	0.12 (1/818)	0.993
Anaphylaxis	0.12 (1/827)	0.12 (1/820)	0.997
**Treatment for adverse reactions**	0.12 (1/827)	0.37 (3/818)	0.311

Data are shown as the percentage (number of applicable women/number of women targeted for analysis). *χ2 test test

The number of subjects is calculated by excluding the missing values from the total number of each group

### Reasons for not receiving mRNA vaccination

Table 5 lists the reasons why postpartum women did not receive an mRNA vaccine during pregnancy during the COVID-19 pandemic. Responses were obtained from 195 of the 217 women. More than 90% of these women were concerned about the impact of vaccination on their fetuses. In addition, more than half of the women were concerned about the impact of vaccination on themselves. Approximately 15% of the women did not wish to be vaccinated regardless of their pregnancy status. Some women were not vaccinated due to vaccine stock-outs during the study period.

**Table 5:** Reasons for not receiving mRNA vaccination (*N* = 195)

Anxiety about the effects on the fetus %	90.8 (177/195)^a^
Anxiety about the effects on the mother %	54.4 (106/195)^a^
No hope for vaccination %	15.4 (30/195)^a^
Wanted to get vaccinated but could not %	7.2 (14/195)^a^

Data are shown as the number or percentage (number of applicable women/number of women targeted for analysis)

^a^Including multiple answers

## Discussion

In this study, we assessed the status of COVID-19 mRNA vaccination, COVID-19 prevalence, and perinatal outcomes among pregnant Japanese women. Approximately 80% of the pregnant women had received at least one dose of vaccination against COVID-19. A high proportion of pregnant women who did not receive an mRNA vaccine had a history of asthma and food or drug allergies. The vaccinated group had a significantly lower incidence of SARS-CoV-2 infection and COVID-19-related pneumonia than the unvaccinated group. No association was observed between mRNA vaccination and adverse perinatal outcomes or the incidence of congenital diseases, including malformations. Adverse reactions were more frequent with the second dose of the mRNA vaccine than with the first dose, although no significant difference was noted in the incidence of serious adverse reactions according to the dose. More than 90% of pregnant women who did not receive an mRNA vaccine during pregnancy were concerned about its potential adverse effects on the fetus; more than 50% were concerned about the effects on themselves, and 15% were unwilling to receive a COVID-19 vaccine. Notably, a small number of pregnant women could not be vaccinated even if they wanted to.

The proportion of pregnant women with a history of asthma and allergies was higher in the vaccinated group than in the unvaccinated group. Given that asthma and allergy have similar background factors, some women may not have been vaccinated because of concerns about possible allergic reactions against mRNA vaccination. Although the incidence of allergies due to mRNA vaccines is very low, 11.1 of 1 million people receiving the BNT162b2 vaccine develop an allergic reaction [16]. One prospective cohort study found that the incidence of allergy increased among those at particularly high risk of allergy after receiving the BNT162b2 vaccine [17]. In one case report from Japan, a patient developed life-threatening acute asthma exacerbation after BNT162b2 vaccination [18]. As the risk of allergic reactions is higher in individuals with a history of allergies than in individuals without a history of allergies and as patients with asthma may be at increased risk as well, the advantages and disadvantages of vaccinating high-risk individuals should be fully explained to the patients to support their decision-making process.

The present study included a cross-sectional design, and although it was difficult to verify the results of vaccine efficacy, previous reports [4, 5, 19] have shown that mRNA vaccinations are effective in reducing both the incidence and severity of COVID-19. However, SARS-CoV-2 undergoes frequent mutation. Even if the current vaccines were effective against the then prevalent strains in Japan (Delta, BA.1; or Omicron, BA.2) [11], it is unclear whether similar results would be obtained against other variants that are associated with reduced neutralizing antibody activity [20, 21]. Further research is necessary to determine the effectiveness of vaccination against other variants. It is critical to collect information on each prevalent variant, vaccine type, and vaccination coverage by country and region.

Consistent with the findings of a previous study [22], birth weight, and length at birth did not differ between the vaccinated and unvaccinated groups in our study. No neonatal deaths were reported in the vaccinated group. A previous study found that mRNA vaccination during pregnancy did not affect the incidence of congenital diseases, including malformations [22]. Similarly, in women in the vaccination group in our study, vaccination showed no effect on the incidence of congenital diseases, including malformations, during the first trimester of pregnancy, and the incidence was similar to that of the general population in both groups [23]. In this study, the preterm delivery and cesarean section rates in the vaccinated group were lower than those in the unvaccinated group. The preterm delivery rate among patients with COVID-19 in the unvaccinated group was slightly higher than that of the entire unvaccinated group (17.6% and 13.7%), but the cesarean section rate was rather low (41.2% and 42.2%). Moreover, it was challenging to establish a causal relationship between the higher COVID-19 infection rates observed in the non-inoculated group and the higher rates of preterm delivery or cesarean section in this study. On the other hand, high levels of anxiety are reportedly associated with premature birth and increased cesarean section rates [24, 25]. Vaccination for SARS-CoV-2 infection is reportedly associated with a reduced risk of anxiety and depressive symptoms [26], possibly contributing to the reassurance that vaccination protects the mother from SARS-CoV-2 infection. However, as the study design and subject matter differ from the previous study, there remains some uncertainty as to the validity of the hypothesis. Moreover, preterm birth and cesarean section rates are associated with several factors, and it is unclear why these rates were lower in the vaccinated group than in the unvaccinated group in our study. Further research is needed in this regard in the future.

Our study confirmed that the overall incidence of adverse effects due to mRNA vaccines was higher after the second dose than after the first dose, as reported previously for the general population and pregnant women [2, 27, 28]. A few reports have stated that myocarditis may occur as a severe adverse effect of mRNA vaccination [29]. In this study, no severe adverse effects of vaccination were reported, confirming that there are no major safety concerns regarding mRNA vaccines. In Japan, three or more vaccinations are now being administered as boosters; however, the effects of receiving additional doses of vaccine on perinatal outcomes, the frequency of adverse reactions in pregnant women, and the long-term effects during pregnancy on the fetus are not fully understood. As observed in this study, pregnant women are hesitant to receive the vaccine mainly because of concerns about adverse effects on the fetus. Large-scale studies investigating not only the short-term effects on the fetus but also the long-term effects are needed.

This study has some limitations. The study was retrospective in nature, and the number of participants was relatively small despite the multicenter study, and some data were missing. Furthermore, the detailed clinical course, including indications and reasons for premature births, and the precise period between vaccination and SARS-CoV-2 infection are unknown. Participating facilities were mainly university hospitals around Tokyo, which care for many high-risk pregnant women; therefore, women with high-risk pregnancies may have been over-represented, and this may explain the relatively high cesarean section rate. At the time that the protocol was developed, the policy was to administer two doses of mRNA vaccine in Japan; however, during the study, the circumstances changed, and three or more doses of vaccinations were being recommended. Despite these limitations, our study provides critical data on the current epidemic caused by the SARS-CoV-2 Delta and Omicron variants and, to the best of our knowledge, this is the first study to report the perinatal outcomes of women who received mRNA vaccination during pregnancy in Japan.

## Conclusions

Our study sheds light on vaccination coverage, perinatal outcomes, timing, and adverse effects and short-term safety of mRNA vaccination and shares details on concerns regarding COVID-19 vaccination among pregnant women in Japan. In this study, mRNA vaccines showed no adverse effects on short-term perinatal outcomes but might have prevented SARS-CoV-2 infection or COVID-19 severity. Hence, mRNA vaccines can be considered safe and effective for use in pregnant women. However, because many pregnant women worry about the adverse effects on the fetus, it is necessary to conduct further research to confirm not only the short-term safety but also the long-term safety of mRNA vaccines.

## Supplementary Information

**Additional file 1.** Distribution of the 15 participating  institutions.

**Additional file 2.** Distribution of the percentage  of deliveries in each gestational  week in the vaccinated  and unvaccinated groups.

## Data Availability

The datasets used and/or analyzed during the current study are available from the corresponding author on reasonable request.

## Abbreviations

COVID-19: Coronavirus disease 2019
SARS-CoV-2: Severe acute respiratory syndrome coronavirus 2
