# Supplementary figures and images for: COVID-19 mRNA vaccination status and concerns among pregnant women in Japan: a multicenter questionnaire survey
Source: BMC Pregnancy Childbirth. 2023 May 9;23:332. doi: 10.1186/s12884-023-05669-4 (PMC10169175; doi:10.1186/s12884-023-05669-4)

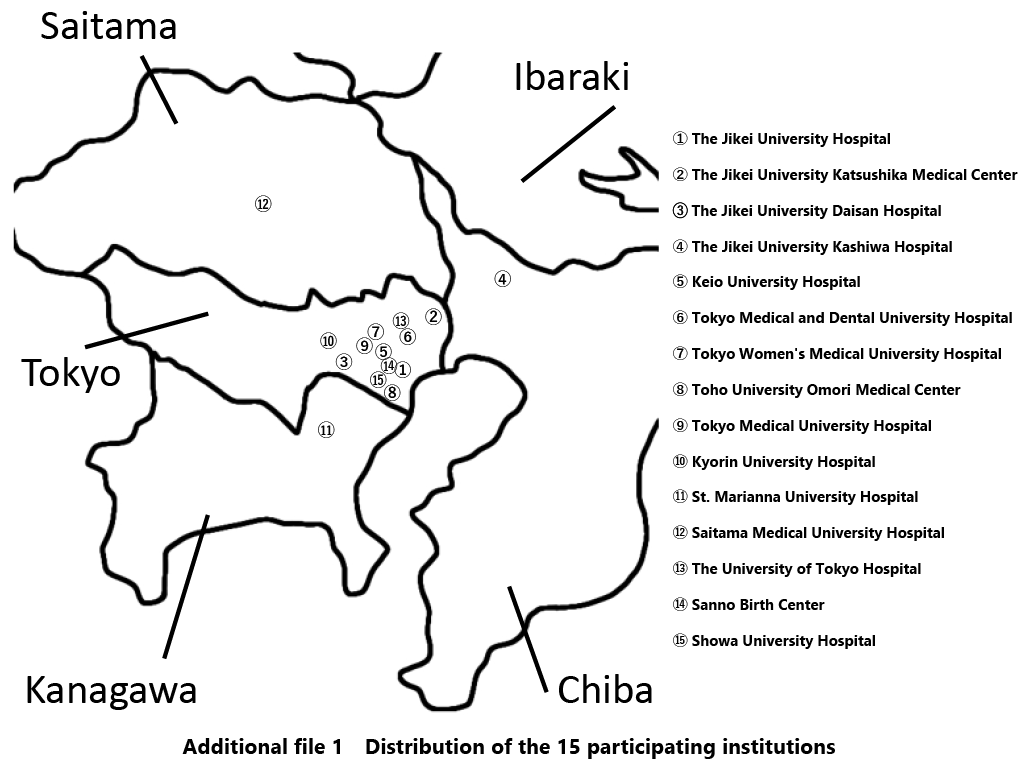

Supplement: Supplementary file 1 — Additional file 1. Distribution of the 15 participating institutions. [file 12884_2023_5669_MOESM1_ESM.tiff]

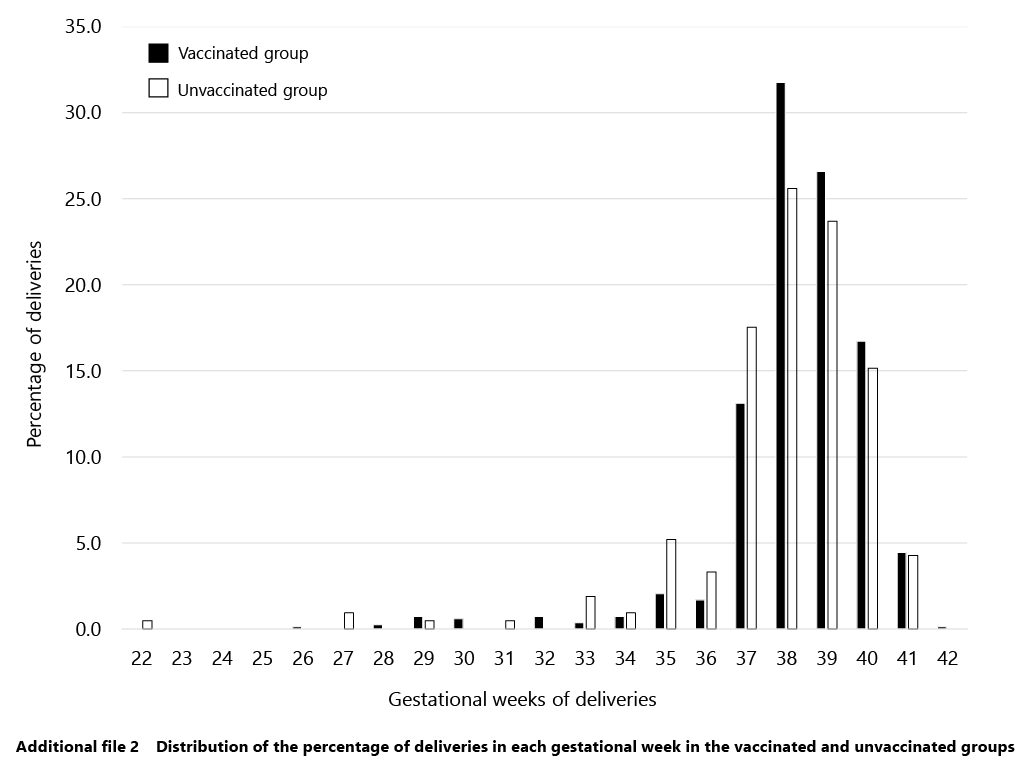

Supplement: Supplementary file 2 — Additional file 2. Distribution of the percentage of deliveries in each gestational week in the vaccinated and unvaccinated groups. [file 12884_2023_5669_MOESM2_ESM.tiff]
